# Endogenous ABA concentration and cytoplasmic membrane fluidity in microspores of oilseed rape (*Brassica napus* L.) genotypes differing in responsiveness to androgenesis induction

**DOI:** 10.1007/s00299-013-1458-6

**Published:** 2013-06-05

**Authors:** Ewa Dubas, Franciszek Janowiak, Monika Krzewska, Tomasz Hura, Iwona Żur

**Affiliations:** Institute of Plant Physiology, Polish Academy of Sciences, ul. Niezapominajek 21, 30-239 Kraków, Poland

**Keywords:** Abscisic acid, Androgenesis induction, *Brassica napus* L., Fluorescence polarization, Microspores, Plasma membrane

## Abstract

****Key message**:**

**A better understanding of androgenesis with a focus on the changes in plasma membrane fluidity and endogenous ABA content affecting embryogenesis induction in microspore suspension of** ***B. napus***.

**Abstract:**

Changes in plasma membrane fluidity (MF) and ABA content associated with androgenesis induction were under the study. Both parameters were monitored in microspores of two *Brassica napus* L. genotypes differing in their response to androgenic induction under heat (1 day at 32 °C). MF was assessed by DPH method. ABA content was evaluated by ELISA. Heat caused microspores’ plasma membrane to become more rigid. Lower MF in microspores of ‘DH 4079’ (of high androgenic potential) seems to maintain proper cell protection and leads to efficient embryogenesis induction. Plasma membrane remodelling coincided with changes of ABA content in microspores and in the culture medium in both genotypes. ABA concentration (μM) and ABA content (fmol *per* 10^4^ microspores or pmol g^−1^ FW) were for the first time measured in microspores. ABA concentration (μM) in microspores and in the culture medium (nM) differed significantly for the genotype and the treatment. The interaction between both variables was also significant. In general, ABA content ranged from <3.5 to 87.1 fmol *per* 10^4^ microspores. The highest content of ABA was detected in ‘DH 4079’ microspores at 32 °C. Assuming a mean microspores’ radius of 10 μm, it corresponds to ABA concentration of 2.1 μM. Heat shock resulted in quantum of medium pH reduction (0.1–0.2) and increased levels of ABA in microspores and in the medium of both tested genotypes. However, heat induced increase of ABA content in microspores of non-responsive ‘Campino’ had no clear-cut impact, on androgenesis induction efficiency, which suggests a more complex mechanism of process initiation.

## Introduction

Rapeseed (*Brassica napus* L.) microspore suspensions can be used as a convenient model system for the examination of the physiological response to various stress stimuli at the cellular level (Ferrie and Caswell 2011). Among various stress factors, high temperature, i.e., 32 °C leads to effective induction of microspore embryogenesis by changing the direction of microspore (mcs) development from gametophytic to sporophytic pathway (Custers et al. 1994, 2001; Custers 2003; Touraev et al. 1997, 2000; Joosen et al. 2007; Supena et al. 2008; Wędzony et al. 2009; Dubas et al. 2011). However, among different *B. napus* genotypes, many are non-embryogenic under standard protocols. A poor embryogenic response limits the utility of desirable cultivars in breeding programs, where doubled haploid (DH) technology using isolated microspore culture is the fastest route to complete plant homozygosity in a single step (reviewed in Wędzony et al. 2009). Therefore, the explanation of the physiological background of androgenesis is essential.

Heat stress causes alterations in physical and chemical properties of membrane lipids in living cells, which influences functional properties of the cell membrane (Monvel et al. 2006; Wahid et al. 2007). It has been revealed that temperature can dramatically change the extent of molecular disorder and molecular motion within a lipid bilayer (Orvar et al. 2000; Mejía et al. 1995). The involvement of membrane fluidity in the perception of heat stress remains controversial (reviewed in Los and Murata 2004). Heat stress usually increases membrane fluidity, although some data showed increased plasma membrane rigidity under high temperature (Wahid et al. 2007). Even slight changes in membrane fluidity significantly affect numerous cell functions including growth, solute transport, signal transduction, and membrane-associated enzyme activities. It has been proven that the activity of membrane-bound proteins, such as the translocators of small molecules, ion channels, receptor-associated protein kinases (Hohmann 2003), and sensor proteins (Marczak et al. 2009; reviewed in Los and Murata 2004) is directly affected by heat.

Heat shock modulates endogenous hormone levels with a stress hormone, abscisic acid (ABA), which is particularly significant in counteracting heat effects (Wahid et al. 2007). Induction of ABA accumulation is an important component of thermotolerance, suggesting its involvement in biochemical pathways essential for survival under heat-induced desiccation stress (Maestri et al. 2002). Moreover, in rape ABA affects embryo water uptake (Schopfer and Plachy 1984) and seed germination (McKee and Finchsavage 1989), and additionally, ABA accumulation during cold hardening is prerequisite for frost tolerance acquisition by oilseed rape plants (Smolenska-Smyk et al. 1995).

ABA has also been proposed to be putatively involved in the reprogramming of microspore development (Wang et al. 2000; Zur et al. 2008, 2009). In several reports, enhanced accumulation of ABA was associated with androgenesis induction (Imamura and Harada 1980; Davies and Jones 1991; Van Bergen et al. 1999; Żur et al. 2008, 2009). However, a lack of correlation between ABA level and androgenesis induction efficiency was also postulated (Żur et al. 2012).

Understanding how microspores detect and recognize heat stress signals and how these processes trigger acquisition of embryogenic competence is of high interest and not yet fully understood. The main objective of this project was to analyze cell-autonomous adaptive responses by an estimation of ABA concentration in single cells triggered by stresses to become totipotent. For such studies, microspore suspension seems to be the perfect model, where a huge number of cells at the same stage of development are cultured without the presence of sporophytic tissue. Therefore, we examined the influence of high temperature stress treatment (24 h at 32 °C) on membrane fluidity and ABA pools (content/concentration). The changes were monitored during androgenic induction in microspore culture of oilseed rape (*B. napus* L.) spring genotypes differing in responsiveness to androgenesis induction.

## Materials and methods

### Donor plants and growth conditions

Two spring rapeseed (*B. napus* L.) cultivars: cv ‘Topas’ DH line 4079 (kindly provided by dr. J. Custrers, PRI, Wageningen, Netherlands) and cv ‘Campino’ (received from Norddeutsche Pflanzenzucht, Hans-Georg Lembke KG, Holtsee, Germany) were used in the study.

Cv ‘Topas’ DH 4079 line is a highly embryogenic DH line derived from cultivar ‘Topas’ and commonly used as a model in studies of embryogenesis (Custers 2003). Cv ‘Campino’ was chosen on the basis of preliminary results received in the frame of a Polish-Belgian project (2010–2012) (data not published). The choice of ‘Campino’ was based on the significant difference in the responsiveness to androgenic stimuli (heat shock, 24 h 32 °C) as compared to ‘DH 4079’ line. Moreover, cv ‘Campino’ (registration no. R 1714, 2006–2016) is registered in the Polish National List (NLI) issued by COBORU (http://www.coboru.pl), an official register containing the cultivars of agricultural, vegetable, and fruit plants whose seed material is eligible in Poland. Due to high concentrations of erucic acid and low levels of glucosinolates, it belongs to economically important rape seed cultivars in the industrial oil market (see below the Scheme of the experiment in Table 1).

**Table 1:** Scheme of the experiment. Two spring *B. napus* L. genotypes: cv ‘DH4079’ line and cv ‘Campino’ were grown under greenhouse conditions at 18/18 °C (day/night) temperature until the beginning of bolting. Then plants were divided into two groups: (1) continuing growth at 18/18 °C (day/night) temperature and (2) transferred to continuous mild temperature of 10/10 °C (day/night). After next 10 days, flower buds were excised from donor plants. Some of excised flower buds (2.6–2.8 mm in length) were collected in Eppendorf tubes and frozen in liquid nitrogen. Some of excised flower buds were used for microspores (mcs) isolation. Isolated mcs were cultured at a density of 40,000 ml^−1^ in NLN-13 culture medium. Mcs were cultured at various culture temperature regimes: (1) 18 °C, and (2) 32 ± 0.2 °C for 24 h and then 25 °C

Genotypes of *Brassica napus* L. in tissue/cell	Growth conditions of donor plants	Type of tissue/cells	Measurements			
			Membrane fluidity	ABA content or concentration in tissue/cell	ABA content in NLN-13 medium	pH value in culture medium
‘DH4079’ line/‘Campino’	18/18 °C (day/night)	Flower buds		X		
		mcs on the isolation day		X		
		mcs 1 day onNLN-13 medium at 18 °C		X	X	X
		mcs 1 day on NLN-13 medium at 32 °C		X	X	X
	10/10 °C (day/night)	Flower buds		X		
		mcs on the isolation day		X		
		mcs1lday onNLN-13 medium at 18 °C	X	X	X	X
		mcs 1 day on NLN 13 medium at 32 °C	X	X	X	X

Plants of *B. napus* L., cv ‘Topas’ line DH 4079 and cv ‘Campino’ were grown under greenhouse conditions at 18/18 °C (day/night) temperature at 16/8 h (day/night) regime until the beginning of bolting (about 9 weeks). Then the plants were divided into two groups: (1) continuing growth at 18/18 °C (day/night) temperature and (2) transferred to continuous mild temperature of 10/10 °C with 16/8 h (day/night) photoperiod provided by 150 μmol m^−2^ s^−1^ HPI (Philips) lamps.

In order to measure membrane fluidity, mcs were collected from petri dishes after 1 day at 18 °C (mcs 1 day on NLN-13 medium at 18 °C), and at 32 °C (mcs 1 day on NLN-13 medium at 32 °C).

In order to measure ABA content/concentration in mcs directly after isolation (mcs on the isolation day), after 1 day at 18 °C, and at 32 °C, pellet containing mcs was collected in Eppendorf tubes and frozen in liquid nitrogen immediately before ABA measurements. Endogenous ABA level was also measured in culture NLN-13 medium collected from 1-day-old mcs suspension culture at tested temperature conditions (18 or 32 °C). In addition, the pH value of the culture medium collected from 1-day-old mcs suspension culture was determined at tested temperature conditions (18 or 32 °C).

### Microspore isolation

Microspores were isolated from flower buds (2.6–2.8 mm in length) following the protocol of Custers (2003) with modifications by Joosen et al. (2007).

Modified NLN-13 medium (Lichter 1982) with 13 % sucrose (w/v), without potato extract and growth regulators was used for isolation and culture of mcs. In order to prevent fluctuation in temperature during the isolation procedure, all the solutions used for sterilization were stored at 4 °C and used immediately after being taken out of 4 °C. In order to measure ABA in mcs directly after isolation, pellet containing mcs was collected in Eppendorf tubes and immediately frozen in liquid nitrogen.

### Microspore culture

The pellet containing mcs was resuspended, and mcs were incubated at a density of 40,000 ml^−1^ in NLN-13 culture medium. Aliquots of 3-ml mcs suspension were placed in 6-cm petri dishes for culture and transferred to various culture temperature regimes: (1) 18 °C, where the microspores continued gametophytic development resulting in pollen maturation (Dubas et al. 2012) and (2) 32 ± 0.2 °C for 24 h and then 25 °C, which is a mild heat stress inducing formation of mcs-derived embryos with suspensors (Supena et al. 2008; Dubas et al. 2011).

The progress of mcs-derived embryo development was monitored with light microscopy. Samples were taken at the isolation day and after 1 day of culture at 32 °C or at 18 °C for DAPI staining (4′,6-diamidino-2-phenylindole 1 μg ml^−1^ + 1 % Triton-X100) and observed under fluorescence microscope Eclipse E 600 equipped with digital camera Nikon DS-Ri1.

### Androgenesis efficiency

To evaluate androgenesis induction efficiency, the number of mcs-derived embryos was estimated after 4 weeks of culture and calculated per 10,000 isolated microspores (10^4^). Five repetitions were performed for each cultivar (‘Topas’ DH line 4079, ‘Campino’) and treatment (18 or 32 ± 0.2 °C for 24 h).

### Determination of ABA content

Endogenous ABA level was measured in whole flower buds as well as in microspores isolated from the flower buds of donor plants growing at 18/18 °C and at 10/10 °C at the beginning of flowering with two opened flowers (9-week-old plants). Endogenous ABA level was also measured in culture NLN-13 medium collected from 1-day-old mcs suspension culture at tested temperature conditions (18 or 32 ± 0.2 °C). Microspore suspensions were derived from both treatments of donor plants (18 or 10 °C).

Harvested flower bud tissue was immediately frozen in liquid nitrogen and freeze-dried. The samples were ground with ball mill MM400 (Retch, Germany) in Eppendorf vials, to which 1 mL of cold distilled water was then added. Next the vials were placed in boiling water for 3 min and shaken overnight at 4 °C. The next day the extracts were centrifuged for 20 min in a refrigerated centrifuge at 18,000×*g* (MPW-350R, Poland). ABA was measured in the supernatant using indirect enzyme-linked immunosorbent assay (ELISA) according to Walker-Simmons and Abrams (1991). The antibody used was MAC 252 (Babraham Technix, Cambridge, UK). Absorbance was measured by microplate reader Model 680 (Bio-Rad Laboratories, Inc.) at the wavelength of 405 nm. Material was collected independently for each genotype in three biological replicates. One biological replicate contained flower buds of ca. 0.01 g of FW.

To the samples of microspores 1 ml of cold distilled water was added. Then they were frozen and thawed for better ABA extraction and shaken overnight at 4 °C. The rest of the procedure was the same as for bud samples.

ABA concentration (μM) in mcs was estimated according to the mean diameter of mcs (20 μm). Mcs’ diameter was measured three times for population of 500 microspores collected directly after isolation procedure. Measurements were done under light microscope.

ABA concentration in the samples of the medium was measured after freezing, thawing, and centrifugation as described above.

For each treatment, at least nine independent ELISA measurements were performed on three pooled samples, each collected from five different plants or petri dishes.

### Measurements of membrane fluidity—fluorescence polarization

Membrane fluidity was determined indirectly though the determination of fluorescence polarization of 1,6-diphenyl-1,3,5-hexatriene (DPH) probes within the hydrophobic core of the membrane (Grunberger et al. 1982; Lentz 1989, 1993). For the insertion of the marker, the freshly isolated *microspores* were incubated with 5 μM DPH at subsequent culture conditions (24 h incubation in darkness at 32 °C; heat shock treatment or at 18 °C; control cultures). Working solution (5 μM) of DPH was prepared from 2-mM stock solution of DPH in acetonitrile. The measurements were carried out for DPH labelled cells, in the two possible relative positions of the polarizers in the excitation and emission beam. The *P* values were calculated from the equation by Lakowicz (1999):

*P* = (Ivv − Ivh)/(Ivv + Ivh); where Ivv and Ivh are fluorescence intensities measured (after appropriate background subtraction) with the excitation polarizer oriented vertically and the emission polarizer oriented horizontally.

*P* values are negatively correlated to cell membrane fluidity, thus a high *P* value represents a high structural order or low cell membrane fluidity (review in Los and Murata 2004; Companyó et al. 2007; Shrivastava and Chattopadhyay 2007).

**Fig. 1:**
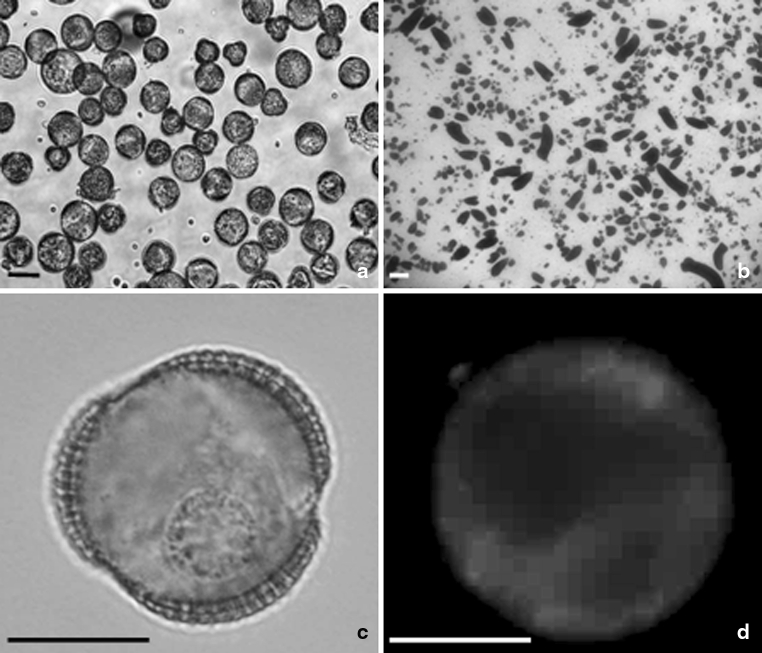
Microspore suspension of *B. napus*. Isolated microspore culture after 1 day at 32 ºC (**a**). Note that the average size of the majority of microspores is 20 μm in diameter. Microspore-derived embryos (*black structures*) of highly embryogenic ‘DH 4079’ line after 4 weeks of culture in NLN-13 medium on Petri dish (**b**). Uni-nucleate microspore after 1 day of heat shock treatment (**c**). Microspore after 1 day of heat shock treatment with incorporated fluorescent dye DPH. Blue fluorescence of DPH in UV light (**d**).* Scale bar*  10 μm (**a**), 1 mm (**b**), and 20 μm (**c**, **d**). Light microscope (**a**–**c**). Fluorescence microscope (**d**)

Fluorescent polarization of DPH was determined with an LS50 spectrofluorometer (Perkin-Elmer Ltd., Beaconsfield, Buckinghamshire, England) using excitation and emission wavelengths of 350 and 430 nm, respectively. The slit widths of both the excitation and emission window were kept at 10 nm. The DPH incorporation was additionally checked on a microscopic slide under UV light using a fluorescent microscope (Fig. 1).

### Measurement of pH value in culture medium

The value of pH was determined in culture medium of *B. napus* microspore suspensions at various temperature growth conditions of donor plants (18/18 °C and at 10/10 °C) and after 1 day of in vitro culture (18 or 32 ± 0.2 °C). The value of pH in medium samples was measured by pH meter pH 211 (Hanna Instruments, Germany) using microelectrode HANHI1330B.

### Light and fluorescence microscopy

Microscopic observations were performed by means of light and fluorescent microscope ECLIPSE-E 600 (NIKON) equipped with Nomarski differential interference contrast (DIC). Images were recorded by digital camera (Digital sight DS-Ri1) and processed by NIS-Elements (AR 2.10 Laboratory Imaging System, Ltd.) program. For visualization of DPH incorporation, analysis was performed under UV light (Exc 330–380 nm, DM 400 nm, BA 420 nm).

### Statistical analysis

The data presented for androgenesis efficiency are means from five replicates (microspore suspensions collected from different petri dishes after heat treatment procedure).

The data presented for membrane fluidity and ABA measurements in microspores and culture medium are means from five replicates (microspore suspensions collected from different Petri dishes; medium collected from microspore suspensions at subsequent days of microspore culture). The evaluation of data started with descriptive statistical analysis (mean ± SE). ANOVA/MANOVA was performed for analyzing the effects of various treatments. Data comparison was based on Duncan test (*P* < 0.05). All statistical analyses were carried out using STATISTICA 10.0 (StatSoft, Inc., USA) software package.

## Results

### Androgenesis induction effectiveness

Two *B. napus* cultivars (‘DH 4079’ line and ‘Campino’) were chosen due to their different response to androgenesis induction.

Low temperature pre-treatment of donor plants was a prerequisite to androgenesis induction. The application of heat shock only was not able to induce androgenesis in microspore suspension derived from plants growing under optimal physiological conditions (18 °C). In that case microspores continued gametophytic development and mature pollen was formed in suspensions after 7 days of culture (data not presented).

Androgenesis induction was obtained only in microspore suspension isolated from low temperature pre-treated (10 °C) donor plants which were subsequently treated with heat shock (1 day at 32 °C). After an additional 13 days of culture at 25 °C, embryogenic structures (mcs-derived embryos) resembling zygotic embryos with suspensors were formed. ‘DH 4079’ line manifested high androgenic induction (mean 2 % mcs-derived embryos per petri dish containing 40,000 ml^−1^ mcs). In contrast, in the same culture conditions, the efficiency of androgenesis induction for cv ‘Campino’ was very low (mean 0.2 % mcs-derived embryos per petri dish containing 40,000 ml^−1^ mcs).

### Membrane fluidity

Heat shock treatment (1d 32 °C) of microspores triggered changes in membrane fluidity.

**Fig. 2:**
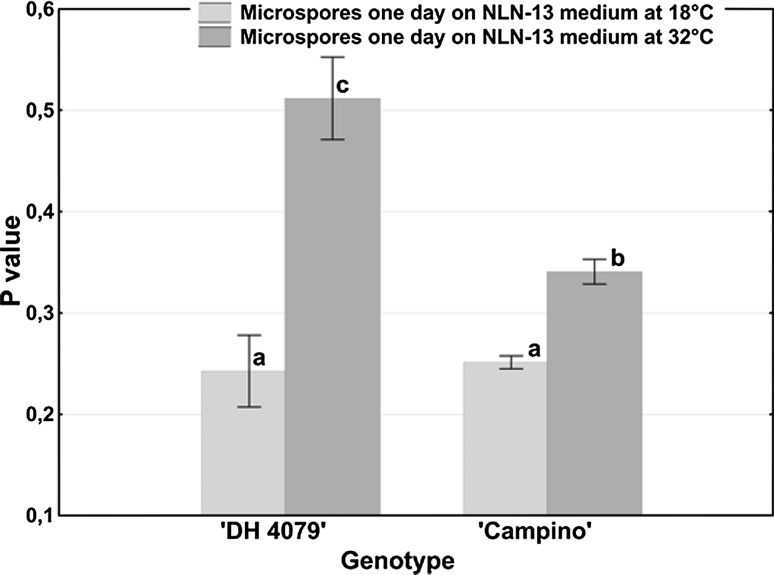
Plasma membrane fluidity (*P* value) in *B. napus* microspore suspensions dependent on the temperature of culture incubation. The fluidity of plasma membranes was estimated using fluorescence dye DPH. Mean values ± standard errors are presented in the graph. Mean values marked with the same letter do not differ significantly according to Duncan’s multiple range test (*P* < 0.05)

The analysis was performed through the evaluation of changes in fluorescence polarization (*P* value) of bound-to-membrane DPH. For both genotypes studied, plasma membranes of microspores cultured for 1 day at 32 °C were relatively rigid in comparison with membrane fluidity of microspores cultured for 1 day at 18 °C (Fig. 2).

The mobility of the DPH fluorescence probe in the membrane was different for both tested cultivars regardless of in vitro culture conditions (1 day at 18 °C or 1 day at 32 °C). The mean value of P was significantly higher for ‘DH 4079’ line microspores as compared to ‘Campino’ microspores.

### ABA level

**Fig. 3:**
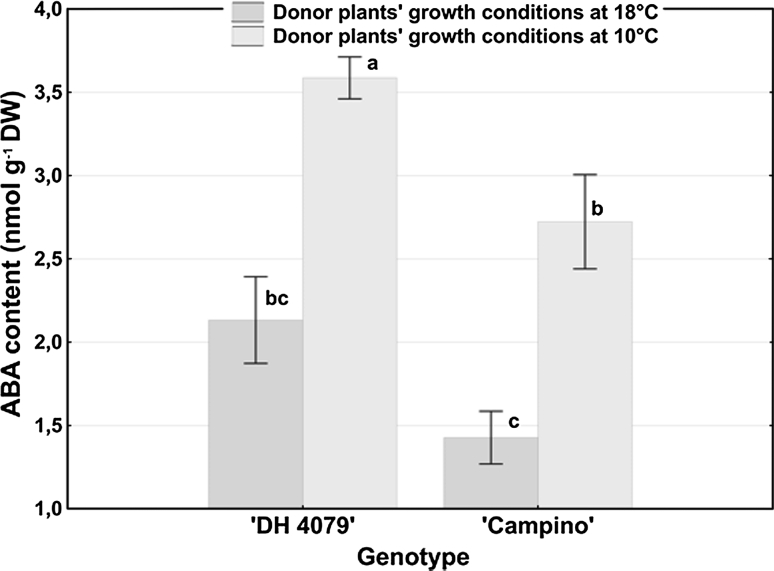
ABA level in *B. napus* flower buds dependent on the temperature of donor plants’ growth conditions. ABA concentration was estimated using ELISA test. Mean values ± standard errors are presented in the graph. Mean values marked with the same letter do not differ significantly according to Duncan’s multiple range test (*P* < 0.005)

**Fig. 4:**
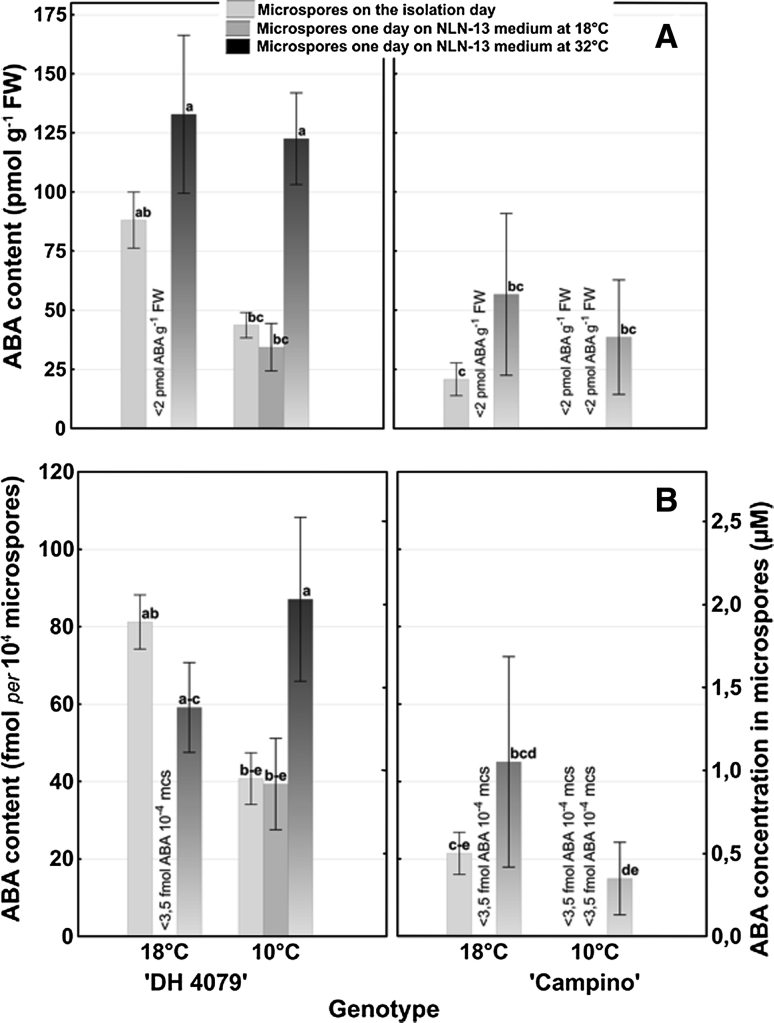
ABA content (pmol g^−1^ FW (**a**) or fmol per 104 microspores (**b**, Y1)) and ABA concentration (μM (B, Y2)) in *B. napus* microspore suspensions dependent on the temperature of donor plants’ growth conditions (18 and 10 °C) and of in vitro culture (18 and 32 °C). ABA content was estimated using ELISA test. Mean values ± standard errors are presented in the graph. Mean values marked with the *same letter* do not differ significantly according to Duncan’s multiple range test (*P* < 0.05)

Endogenous ABA concentration was modulated under stress conditions in flower buds as well as in microspore suspensions (Figs. 3, 4).

### ABA levels in flower buds

Statistical analysis (Fig. 3) revealed that under normal physiological conditions (18 °C) ABA concentration in flower buds did not differ for the tested cultivars.

Transfer of the donor plants to 10 °C was accompanied by an increase in ABA concentration in flower buds for both tested genotypes. The level of ABA significantly increased 1.7- and 1.9-fold in ‘DH 4079’ line and ‘Campino’, respectively. Despite higher ABA accumulation in flower buds of cv ‘Campino’ in response to low temperature, the final concentration of ABA was significantly lower in comparison with ‘DH 4079’ line.

### ABA levels in isolated microspores

Statistical analysis revealed that ABA content (pmol g^−1^ FW, Fig. 4a; fmol *per* 10^4^ mcs, Fig. 4b) and ABA concentration (μM) (Fig. 4b) in isolated mcs differed significantly for genotype and treatment. Significant interaction between both variables was also observed.

In general, ABA content ranged from <3.5 to 87.1 fmol *per* 10^4^ mcs. The highest content of ABA (87.1 fmol *per* 10^4^ mcs) was detected in ‘DH 4079’ mcs at 32 °C. Assuming a mean microspore radius of 10 μm, it corresponds to ABA concentration of 2.1 μM (Fig. 4b).

Under optimal donor plant growth conditions (18 °C), ABA level in mcs on the isolation day was fourfold higher in the responsive ‘DH 4079’ line as compared to ‘Campino’ (Fig. 4a–b). One day of in vitro mcs culture at 18 °C resulted in a reduction of ABA content. Depending on the tested genotype, ABA level decreased 20-fold and almost 90-fold for ‘Campino’ and ‘DH 4079’ line, respectively. Conversely, 1 day of heat shock stress (32 °C) increased ABA levels. The accumulation of ABA was three times higher for line ‘DH 4079’ than for ‘Campino’ as compared to ABA level on the isolation day (Fig. 4a, b).

In mcs suspension isolated from low temperature (10 °C) pre-treated donor plants, ABA level in microspores on the isolation day was significantly lower than in mcs isolated from plants growing at 18 °C. This difference ranged from 2- to 20-fold for ‘DH4079’ line and cv ‘Campino’, respectively (Fig. 4a, b). The response of tested genotypes to in vitro culture conditions was similar to that of above-mentioned microspores isolated from donor plants growing at 18 °C (Fig. 4a, b).

### ABA levels in microspore suspension culture medium

**Fig. 5:**
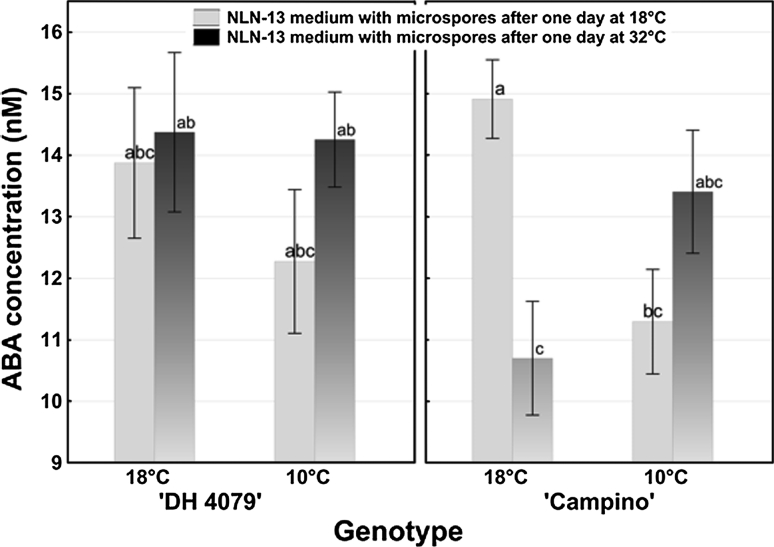
ABA concentration in culture medium of *B. napus* microspore suspensions dependent on the temperature of donor plants’ growth conditions (18 and 10 °C) and of in vitro culture (18 and 32 °C). ABA concentration was estimated using ELISA test. Mean values ± standard errors are presented in the graph. Mean values marked with the *same letter* do not differ significantly according to Duncan’s multiple range test (*P* < 0.05)

ABA concentration in the medium of petri dishes in which microspores were incubated varied from 10.7 to 14.9 nM (Fig. 5). The only significant difference in ABA concentration was observed between culture media collected from microspore suspensions of ‘Campino’ isolated from plants growing at 18 °C and cultured for 1 day at 18 °C and those cultured for 1 day at 32 °C (Fig. 5). After 1 day of treatment at 32 °C, ABA concentration was significantly (1.4-fold) lower in comparison with ABA level in medium collected after 1 day at 18 °C (Fig. 5).

### pH of microspore suspension culture medium

**Fig. 6:**
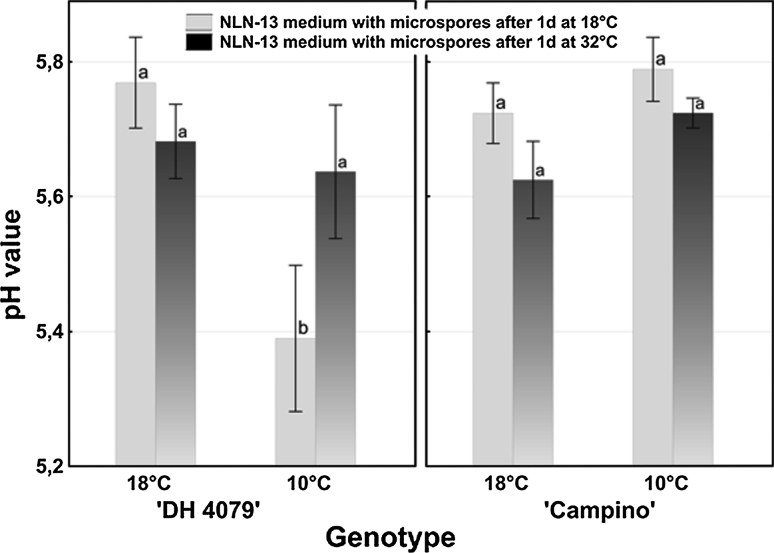
pH value in culture medium of *B. napus* microspore suspensions dependent on the temperature of donor plants’ growth conditions (18 and 10 °C) and of in vitro culture (18 and 32 °C). pH value was estimated using microelectrodes. Mean values ± standard errors are presented in the graph. Mean values marked with the *same letter* do not differ significantly according to Duncan’s multiple range test (*P* < 0.05)

The only significant difference in pH value of NLN-13 medium was observed between culture media collected from microspore suspensions of ‘DH 4079’ line isolated from plants growing at 10 °C and cultured for 1 day at 18 °C and those cultured for 1 day at 32 °C (Fig. 6). One day of treatment at 18 °C of ‘DH 4079’ line mcs caused medium acidification to pH 5.39 ± 0.11 as compared to the initial pH value at the moment of culture set-up (pH 5.8).

## Discussion

The experiments reported here have produced interesting observations on the role of ABA in cold and heat stress responses in flower buds and in isolated single cells (mcs). Both ABA concentration and plasma membrane fluidity were tested in the context of the response to heat stress, which was used as the main trigger in androgenesis induction.

Responses to heat stress vary among plant species and genotypes, type of tissues, and developmental stage (Queitsch et al. 2000). Genotypic variation in plant responsiveness to high temperature stress is a phenomenon characteristic also for various types of androgenic cultures (for review see Powell 1990; Żur et al. 2008, 2009; Wędzony et al. 2009). Isolated mcs are a unique system, where high temperature stress treatment significantly increases the efficiency of androgenesis of many crop species and cultivars (Touraev et al. 1997, 2000). Among *Brassica* genotypes, *B. napus* cv ‘Topas’ embryogenic line ‘DH 4079’ is one of the most responsive genotypes (Ferrie 2003). Some other genotypes of *B. napus* are poorly embryogenic, and only a small part (up to 0.5 %) of cultured mcs form embryos using the standard *B. napus* protocol for microspore embryogenesis including heat shock (30–35 °C) treatment (Ferrie et al. 2005).

We assumed that the differences in responsiveness to androgenesis between ‘Campino’ and ‘Topas’-derived ‘DH 4079’ plants may be related to the responses to stress conditions such as membrane modulation and/or endogenous hormone levels.

As ABA is involved in the regulation of many physiological properties by acting as a signal molecule (Larkindale and Knight 2002), it could play a role in microspore’s ability to respond to temperature stress appropriately. ABA induction is an important component of thermotolerance, suggesting its involvement in biochemical pathways essential for survival under heat-induced desiccation stress (Maestri et al. 2002). For that reason, ABA as a stress hormone was profiled in flower buds, mcs of two studied oilseed rape genotypes, as well as in the culture medium.

The range of ABA concentrations measured in flower buds (10^−9^ to 10^−8^ M ABA g^−1^ DW) of responsive and recalcitrant *B. napus* genotypes was similar to those described by Shukla and Sawhney (2006) or by Malik et al. (2008).

For the first time, isolated mcs were used as a target for endogenous ABA level estimations. ABA concentration in isolated mcs was significantly lower than in flower buds (10^−11^ to 10^−12^ M g^−1^ FW). These results are comparable with ABA concentration (μM) in leaf apoplast (Wilkinson and Davies 2002). Based on the number of isolated cells, we were also able to estimate ABA content in mcs (3.5–87 fmol *per* 10^4^ mcs). Moreover, based on the mean diameter (20 μm) of mcs, we were able to calculate ABA concentration (μM) in a single cell and defined the correspondence of ABA content to ABA concentration (μM).

Heat shock stress resulted in increased levels of ABA in mcs of both tested genotypes (1.4- to twofold for responsive and 0.5- to 15-fold for recalcitrant genotypes). However, the lack of any significant difference between ABA concentration in heat-treated mcs derived from plants growing at 18 °C and at 10 °C (a prerequisite for androgenesis induction) reveals the lack of a direct relation between ABA accumulation and androgenesis induction efficiency. ABA accumulation seems to be a necessary but not sufficient factor for higher androgenesis induction efficiency. Such results are in agreement with data obtained by Żur et al. (2012) in triticale.

Based on the results obtained so far, we can conclude that ABA is involved in a mechanism which is a part of heat stress adaptation (review in Wahid et al. 2007). An increased ABA concentration could sustain mcs viability through an improvement of their tolerance or adaptation to high temperature stress in vitro conditions (Imamura and Harada 1980). It was postulated that the protective ABA function consists of cell membrane stabilization and the initiation of a defence reaction against oxidative stress (Prasad et al. 1994).

The effects of high temperatures on the physical state of membranes have been intensively studied in plants (Vigh et al. 1998; Queitsch et al. 2000). It was shown that heat stress affects plant growth through its ontogeny, and tolerable heat threshold level varies considerably at different developmental stages and depends on the species (Wahid et al. 2007).

In the present study, *P* values for heat-induced embryogenic mcs (culture at 32 ºC) increased as compared to non-induced mcs (culture at 18 ºC), which continued gametophytic developmental pathway. According to the literature, such an increase in *P* value is characteristic for a decrease in cell membrane fluidity (review in Los and Murata 2004; Companyó et al. 2007; Shrivastava and Chattopadhyay 2007).

We found that plasma membrane in microspores of ‘DH 4079’ line was modulated under heat shock and its fluidity was significantly different in comparison with ‘Campino’. As line ‘DH 4079’ is responsive to androgenesis induction under heat temperature conditions, observed plasma membrane modulations are supposed to maintain proper cell protection against stress connected with microspore embryogenesis induction treatment. According to the majority of research, high temperatures modulate membrane fluidity to prevent disintegration of the membrane lipid bilayer (review in Los and Murata 2004).

In *Arabidopsis* exposed to high temperatures the ratio of unsaturated to saturated fatty acids decreases (Somerville and Browse 1991). An increase in saturated fatty acids of membranes increases their melting temperature and induces heat tolerance.

However, some reports present opposite data. It was determined that an increase in saturated fatty acids (lower fluidity) in mature leaves of maize reduced heat tolerance of the plant (Karim et al. 1997). It should be noted, however, that in some species heat tolerance does not correlate with the degree of lipid saturation, suggesting that factors other than membrane stability might be limiting the growth at high temperatures (review in Wahid et al. 2007).

An increase in the rigidity of the membranes optimizes the dynamics of membrane-associated processes during heat shock (Vígh et al. 2005). Our findings from isolated microspores of *B. napus* are consistent with previous reports in *Arabidopsis thaliana*, where similar changes in plasma membrane fluidity were observed when growth temperature was shifted above 30 °C (Falcone et al. 2004). This suggests that the mechanisms leading to the remodelling of membrane lipids during temperature acclimation are conserved across some plant species.

We found a relation between the physical property of microspores’ membrane (P, fluidity), the pH value and ABA concentration in NLN-13 medium collected from ‘DH 4079’ microspore suspensions under subsequent temperature conditions (1 day at 18 °C or 1 day at 32 °C). High ABA concentration in culture medium was associated with higher endogenous ABA content within microspores and their plasma membrane rigidization on less acidic medium pH (above 5.6) at 32 °C. It can be explained based on the ‘anion trap’ concept. As a weak acid (pKa = 4.8), ABA is mostly uncharged when present in a relatively acidic environment and can easily cross cell plasma membrane (Finkelstein and Rock 2002). Intensively produced endogenous ABA in isolated mcs under heat stress conditions migrates across plasma membrane towards alkaline medium environment, where it dissociates to non-transportable ABA anion. Lower ABA content in mcs cultured at 18 °C coincides with a relatively high ABA concentration in culture medium (pH < 5.4) because almost all ABA is redistributed bi-directionally across relatively fluid plasma membrane.

## Conclusions

Based on the present study, microspore suspension of *B. napus* could be proposed as a promising new model for single cell signalling studies towards a better understanding of stress tolerance and abscisic acid action.

It can be concluded that a low ABA level coincides with higher plasma membrane fluidity, which could be the cause for the failure of microspore embryogenesis induction. Heat-induced increase of ABA content seems to be a prerequisite for efficient androgenesis induction. However, no direct link between ABA concentration/content and androgenesis induction efficiency was revealed. It suggests a more complex, multifactorial mechanism of androgenesis initiation with ABA as one among its many components. The precise role of ABA in the context of plasma membrane modulation still remains to be elucidated.

## Abbreviations

ABA: Abscisic acid
DHs: Doubled haploids
DPH: 1,6-diphenyl-1,3,5-hexatriene
DW: Dry weight
ELISA: Enzyme-linked immunosorbent assay
FW: Fresh weight
MC: Microspore culture
Mcs: Microspores
P: Fluorescence polarization
T-Test: Student statistical test
